# Development of a Questionnaire to Assess the Psychosocial Effects of COVID-19 on Peripartum Women

**DOI:** 10.7759/cureus.14270

**Published:** 2021-04-03

**Authors:** Archana Kumari, Keerthana Rajasekaran, Piyush Ranjan, Ashish D Upadhyay, Anju Singh, Rakesh Kumar Chadda, Neerja Bhatla

**Affiliations:** 1 Obstetrics and Gynaecology, All India Institute of Medical Sciences, New Delhi, New Delhi, IND; 2 Medicine, All India Institute of Medical Sciences, New Delhi, New Delhi, IND; 3 Statistics, All India Institute of Medical Sciences, New Delhi, New Delhi, IND; 4 Psychiatry, All India Institute of Medical Sciences, New Delhi, New Delhi, IND

**Keywords:** covid-19, psycho- social functioning, behavior, pregnancy, peripartum, questionnaire

## Abstract

Objective

The social and behavioral changes brought about by the coronavirus disease 2019 (COVID-19) pandemic have led to adverse consequences on the psychosocial functioning of peripartum women. Therefore, we developed and validated a tool to assess the psychosocial effect of the pandemic among these women.

Methods

The questionnaire was developed using a scientifically accepted systematic methodology comprising literature search, focus-group discussion (FGD), expert evaluation, pretesting, and validation.

Results

The final questionnaire consists of 38 questions, and it has a Cronbach's α value of 0.90 and a Kaiser-Meyer-Olkin (KMO) value of 0.773.

Conclusion

The questionnaire has good reliability and face, content, and construct validity. It can be used to assess the psychosocial functioning of peripartum women in low middle-income countries and help perinatal mental health specialists to devise strategies to cope with the psychological impact of COVID-19-like pandemics on peripartum women.

## Introduction

The coronavirus disease 2019 (COVID-19) pandemic has had pervasive psychosocial effects, significantly affecting various sections of society. The peripartum phase (the phase comprising late pregnancy and early postpartum periods) is considered to be a life-changing period among women, and it is characterized by significant physiological and psychological changes. Recent studies have reported that 10-20% of peripartum women suffer from mental health problems, with its prevalence observed more in the low middle-income countries [1,2]. The emergence of pandemics such as COVID-19 with its associated alterations in societal norms can also affect the psychological health of peripartum women. Home quarantine and social withdrawal have upended the psychological and social life of these women [3,4]. The inability to visit doctors, meet family/friends, and attend social and religious gatherings may have adverse consequences on the psychosocial functioning of these women [5]. Moreover, the fear of contracting COVID-19 and its possible effects on pregnancy and unborn/newborn may have a negative impact on these women [3]. A study by Ostacoli et al. (2020) found an elevated risk of the development of maternal postpartum depression and anxiety during the COVID-19 pandemic [6].

Mental health problems are known to have negative effects on maternal, neonatal, and infant outcomes, leading to conditions such as preeclampsia, antepartum and postpartum hemorrhage, fetal growth impairment, preterm births, and stillbirths [7,8]. During the ongoing COVID-19 pandemic, there have been reports of increased risk of preterm births, delayed cognitive and emotional development of infants, and reduced parent-child bonding [9]. This issue is of major concern and it is imperative to assess the effect of the pandemic on peripartum women in various societies. Recently, many studies conducted worldwide have assessed the psychosocial impact of COVID-19 on peripartum women using various validated tools such as the Edinburgh Depression Scale (EDS), Generalized Anxiety Disorder 7‐item Scale (GAD‐7), Impact of Event Scale-Revised (IES-R), Beck Depression Inventory-II, and the State-Trait Anxiety Inventory [6,10,11]. However, these tools may not be uniformly applicable to all population groups including peripartum women. Hence, this study aimed to develop and validate a questionnaire suitable for the Indian population based on its unique socio-cultural makeup to assess the psychosocial effects of COVID-19 on peripartum women.

## Materials and methods

The primary objective of the study was to develop and validate a questionnaire to assess the psychosocial effects of COVID-19 on peripartum women. The questionnaire was developed using a scientifically accepted systematic methodology comprising literature search, focus-group discussion (FGD), expert evaluation, pretesting, and validation [12]. The study was approved by the Institute Ethics Committee at AIIMS, New Delhi (IEC/549/6/2020), and informed consent was received from all participants prior to the study initiation.

Study design

A mixed-method approach for questionnaire development and validation was used, which consisted of two phases.

Phase 1: Questionnaire Development

**Figure 1:**
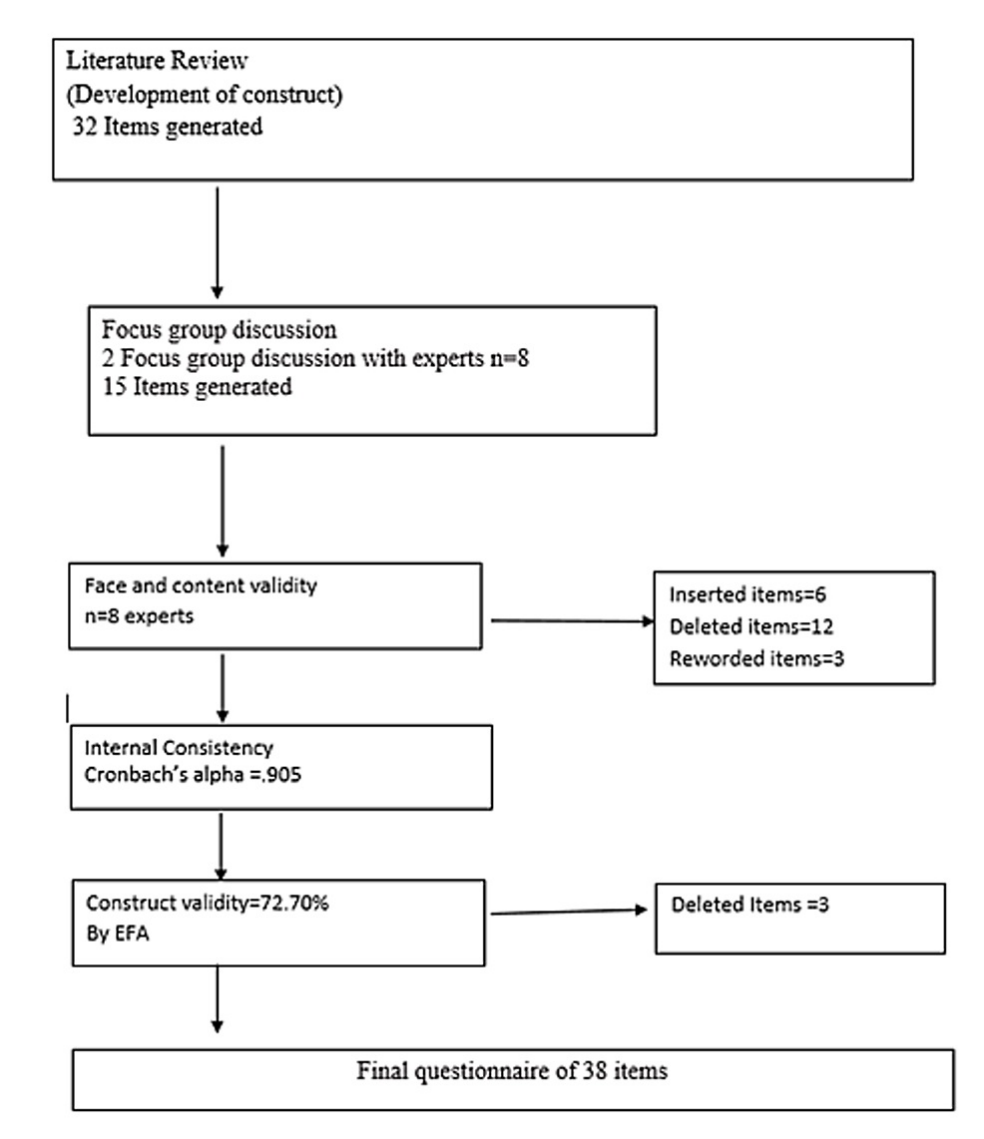
Flow chart illustrating questionnaire development and validation EFA: exploratory factor analysis

Questionnaire development was done using a systematic methodology of four main steps: literature review, FGD, expert evaluation, and pilot testing as summarized in Figure 1.

Literature review: an in-depth literature search was done using the electronic search engines Google Scholar and PubMed. Keywords such as “coronavirus”, “pandemic”, “COVID-19”, “scale”, “questionnaire”, “tool”, “psychological”, “social”, “behavioral”, “pregnant”, “postpartum”, “peripartum” were used to identify relevant studies. The initial literature search returned 54 papers. After screening the titles, abstracts, and full texts, 17 relevant studies were identified and read through for the purpose of item generation. Thirty-two items were generated in this step.

FGDs and in-depth interviews: three FGDs constituting five participants in each group were conducted, two in the antenatal ward and one using an online platform. Each discussion lasted for around 45-50 minutes. In addition, 10 in-depth interviews were also conducted, each session lasting for 20-25 minutes. Anonymity and confidentiality of participants were ensured. The data obtained was analyzed qualitatively. The relevant psychological and social domains were identified [3]. This step led to the generation of 15 items.

Literature search, FGDs, and in-depth interviews led to the generation of 47 items in total. All items were framed in simple and lucid language, avoiding double negatives, ambiguity, and overlapping. For the response to the items, a 5-point Likert scale was used, assuming equal distance between response objects.

Expert evaluation: the developed questionnaire was critically evaluated for its face and content validity by a team of eight experts from the Departments of Obstetrics and Gynaecology, Medicine, Clinical Psychology, and Psychiatry. On the basis of their evaluation, six items were added, 12 were removed (due to their non-relevancy), and three were reworded (to enhance their clarity and bring in simplicity).

Pretesting: the evaluated questionnaire was pretested on 15 peripartum women of different ages and educational qualifications to evaluate the comprehensibility and acceptability of the tool. The participants responded to the questionnaire and also commented upon the necessity, clarity, relevance, and simplicity of each of the items. In this step, three items were eliminated. Thus, the final version of the questionnaire comprised 38 items.

Phase 2: Validation of the Questionnaire

In this phase, the questionnaire was distributed to 194 peripartum women of different ages, educational qualifications, and socioeconomic status to ensure maximum diversity. The sample size of 194 was determined based on the guidelines related to the respondent-to-item ratio (5:1) [13]. The required sample size was determined to be 190. The data was collected online/offline in December 2020.

Statistical analysis

The face validity and content validity of the developed questionnaire were established through FGDs, expert evaluation, and pretesting. An exploratory factor analysis with varimax rotation was carried out for construct validity to test the domain structure [14]. This technique is used to estimate factors and/or to reduce the dimensionality of a large number of variables to a fewer number of factors. The Cronbach's α was used to assess internal consistency (i.e., the extent to which the items on the instrument measured the same thing). A Cronbach α value of 0.7 or higher was considered to indicate good internal consistency [15]. The data were analyzed using IBM SPSS Statistics software v24 (IBM, Armonk, NY).

## Results

The final questionnaire, approved by experts and pilot-tested on peripartum women for its content and face validity, has six parts and is freely available for use. Section A of the questionnaire comprises general information and demographic data. Section B has 38 questions. Questions 1-9 are related to the emotions and thoughts that peripartum women might have experienced during the COVID-19 pandemic. Questions 10-15 address the various factors responsible for the fear/negative emotions affecting their general well-being. Questions 16-21 deal with the various measures adopted by peripartum women due to fear of getting infected with COVID-19. Questions 22-32 are directly related to the health concerns of peripartum women. Questions 33-38 are about the various coping mechanisms taken up by peripartum women during the COVID-19 pandemic. The impact is scored on three levels: mild (38-63), moderate (64-89), and severe (90-114). The questionnaire requires less than 25 minutes to complete. Table 1 presents the socio-demographic profile of the study participants The Appendix section lays out the two sections of the questionnaire as it was provided to the participants (Tables 2, 3).

Socio-demographic profile of the study participants

A total of 194 peripartum women participated in the survey for the validation of the questionnaire (Table 1). The mean age of the participants was 29.31 ±3.76 years (range: 20-44 years). Around 81% of them belonged to the middle class.

**Table 1:** Socio-demographic profile of study participants

Characteristics	Frequency (%)
Age, years	
18-24	25 (14.06%)
25-34	160 (82.09%)
35 and above	9 (4.0%)
Parity	
Primigravida	81 (45.9%)
Multigravida	113 (54.1%)
Type of conception	
Spontaneous	192 (98.8%)
Assisted	2 (1.2%)
Pregnant women	88 (45%)
Period of gestation, weeks	
30-33	36 (40%)
34-37 weeks	23 (26%)
>37 weeks	29 (33%)
Postpartum women	106 (55%)
Days following delivery	
Up to 30	84 (79.2%)
More than 30	22 (20.7%)
Mode of delivery	
Spontaneous vaginal delivery	58 (54.7%)
Instrumental	07 (6.6%)
Caesarean	41 (38.6%)
Education	
Up to class X	28 (14.4%)
Up to class XII	62 (31.9%)
Graduation	74 (38.1%)
Post-graduation	30 (15.4%)
Occupation	
Homemaker	136 (70.1%)
Working	58 (29.8%)
Income	
Low	24 (12.3%)
Lower middle	99 (51.0%)
Upper middle	59 (30.4%)
Upper	12 (6.1%)
Medical disorders	
Low risk (uncomplicated pregnancy)	62 (32.0%)
High risk (intrahepatic cholestasis of pregnancy, fetal growth restriction, diabetes mellitus, hypertension, multiple pregnancies, heart disease)	132 (68.0%)

Reliability and validity of the questionnaire

The internal consistency of the questionnaire was α: 0.90. Exploratory factor analysis with the principal component method using varimax rotation was done to establish the tool's construct validity. For determining the number of factors, the criteria of eigenvalues and scree plot were used. Finally, five latent emerged factors explained 72.70% variance and were most appropriate for measuring the psychosocial impact of COVID-19 on peripartum women. Sampling adequacy was confirmed by the Kaiser-Meyer-Olkin (KMO) value, which was 0.773. Bartlett’s test of sphericity was also found to be significant (p = 0.001).

## Discussion

Peripartum is a stressful period requiring significant social support from family, friends, and treating doctors. Perinatal mood and anxiety disorders (PMADs) affect around 15-20% of women and 10% of men respectively [16]. Social factors like lack of support, financial crisis, and illness or loss in family can precipitate PMADs, all of which are more likely to get aggravated due to COVID-19 [17,18].

Pregnant women have a naturally suppressed immune system, making them more vulnerable to infections. During the ongoing COVID-19 pandemic, peripartum women have been identified as one of the most vulnerable groups [19]. The unprecedented effects of COVID-19 on their own health and their babys' health have elevated the levels of maternal anxiety [20,21]. A sense of fear and anxiety about vertical transmission, altered prenatal care modules, misinformation through the news available on various platforms, quarantine measures, and restricted social and religious activities have adversely affected the psychosocial functioning of peripartum women [3]. Loneliness due to social distancing may further amplify the effect of other psychosocial stressors experienced by peripartum women and their families. The adverse psychological impact can further lead to maternal and fetal complications [22,23]. Hence, it is imperative to assess the impact of COVID-19 on the psychosocial functioning of peripartum women.

This study is one of the first efforts in India to develop and validate a questionnaire to assess the impact of COVID-19 on the psychosocial functioning of peripartum women. This questionnaire we developed is comprehensive, user-friendly, and is applicable for Indian peripartum women. The questionnaire comprises 38 questions covering various psychological and social aspects of peripartum women during COVID-19. Items have been generated to assess the emotions experienced by peripartum women during the current COVID-19 outbreak, various factors responsible for the fear/discomfort/negative emotions during the pandemic, concerns related to own health and the health of unborn/newborn infants, and various actions/measures taken to cope with fear/negative emotions during this pandemic.

During this pandemic, researchers worldwide have assessed the psychological impact of COVID-19 on pregnant and postpartum women [6,10,11]. However, the tools used have mainly included the EDS, GAD‐7, IES-R, Beck Depression Inventory-II, and the State-Trait Anxiety Inventory, which might not be uniformly applicable to all population groups. Though these scales are reliable, they have not been modified to specifically assess the psychological impact of the COVID-19 pandemic. On the other hand, there is a paucity of tools to assess the impact due to the social changes. Therefore, we have developed a validated tool that will assist in assessing the impact of COVID-19 on the psychosocial functioning of peripartum women in India as well as other South and Southeast Asian countries.

This questionnaire has several strengths. It is reportedly the first questionnaire exclusively designed to assess the psychosocial impact of COVID-19 on peripartum women among the Indian population. It has good reliability, face, content, and construct validity. It is short, self-administered, and easy to comprehend. However, this study has some limitations. It has an over-representation of sample from the lower middle socioeconomic classes and a predominance of participants from North India. The concurrent/predictive validity of the tool could not be established as it required long-term follow-up.

In conclusion, this validated and reliable tool will help to assess the burden of COVID-19 on the psychological, emotional, and social wellbeing of peripartum women. The information obtained will be useful for obstetricians and policymakers to ensure optimal maternal and infant health in the wake of this current pandemic.

## Conclusions

The questionnaire we have developed has good reliability as well as face, content, and construct validity. It can be used to assess the psychosocial functioning of peripartum women in low middle-income countries and help perinatal mental health specialists to devise strategies to cope with the psychological impact of COVID-19-like pandemics on peripartum women.

## Appendices

Questionnaire: section A 

**Table 2:** General profile UHID: unique health identification number

Name:	Age:	UHID:	
Parity:	Types of conception: spontaneous/assisted		
Whether pregnant or postpartum:			Period of gestation (in weeks):
Postpartum period (in days):			
Mode of delivery (for postpartum): normal vaginal delivery/instrumental/caesarean			
Education: up to class X/XII/graduation/post-graduation			
Occupation: homemaker/working			Family’s monthly income in thousands (Indian rupee)
Whether high-risk pregnancy:			List of medical illnesses (if present)

Questionnaire: section B

This is a 4-point rating scale ranging from 0-3 (0 = not at all, 1 = minimal, 2 = to moderate extent, and 3 = too much). Kindly rate your responses on the basis of the above criteria.

**Table 3:** Questionnaire to assess the impact of COVID-19 on the psychosocial functioning of peripartum women COVID-19: coronavirus disease 2019

	Items	NA	0	1	2	3
	Question numbers 1-9 are related to the emotions and thoughts which you might have experienced in the last few months when you were pregnant or postpartum during the COVID-19 pandemic. Please rate the intensity of these feelings and thoughts that you might have experienced on a scale of 0-3. Please tick anyone of the following options					
1	Fear of getting infected with COVID-19					
2	Fear of family members getting infected with COVID-19					
3	Loneliness due to the COVID-19 pandemic					
4	Fear of losing job/financial loss in business or due to work loss					
5	Frustration with uncertainty due to the COVID-19 pandemic.					
6	Feeling helpless during the COVID-19 pandemic.					
7	Feeling worthless due to the COVID-19 pandemic					
8	Pessimistic (negative) thoughts related to things due to the COVID-19 pandemic					
9	Worry about the future due to the COVID-19 pandemic					
	Question numbers 10-15 mention the various factors responsible for the fear/negative emotions during the COVID-19 pandemic that might have affected your general well-being. How far do you think these factors have affected your general health and wellbeing? Please rate on a scale of 0-3. Please tick anyone of the following options					
10	COVID-19-related news on TV/radio/newspaper					
11	COVID-19-related news on social media like Facebook/WhatsApp					
12	Inability to get support from family, friends, and relatives					
13	Inability to visit the hospital for prenatal/routine check-ups due to the COVID-19 pandemic					
14	The social stigma that if I get COVID-19 infection, all will socially boycott me					
15	Inability to spend quality time with your family and friends (social isolation)					
	During the COVID-19 pandemic, many of us have taken various actions/measures out of fear of getting infected with COVID-19. How far do you think these actions/measures have culminated in discomfort/negative emotions in you? Please rate the same on a scale of 0-3. Please tick anyone of the following options					
16	Avoid the services of domestic help/washerman/driver					
17	Avoiding social gatherings due to the COVID-19 pandemic					
18	Avoiding going to the park for walking/exercising					
19	Avoid using public transport					
20	Avoid eating out/ordering food from outside					
21	Avoiding social ceremonies related to pregnancy (baby shower/Godh Bharai)					
	The next 10 questions are directly related to your care and concern during pregnancy or postpartum. Please rate your response on a scale of 0-3					
22	Avoiding visits to the hospital for prenatal/routine check-ups					
23	To what extent have you been experiencing the fear of complications due to inadequate prenatal services?					
24	How seriously have you been considering taking early maternity leave/break from your job due to the COVID-19 pandemic?					
25	To what extent have you avoided visiting the hospital for prenatal check-ups due to the COVID-19 pandemic?					
26	How much difficulty have you felt in accessing a healthcare facility (meeting doctors/getting scans/going for delivery) during the COVID-19 pandemic?					
27	How much have you been worried about the effect of the COVID-19 on your pregnancy/or on your health?					
28	How much have you been worried about the effect of COVID-19 on your baby?					
29	How determined are you to not hire any additional help for baby care?					
30	How bothered have you been by the effect of a changed lifestyle (diet, exercise, and sleep) during the COVID-19 pandemic?					
31	To what extent have you been making efforts to avoid the thoughts of COVID-19?					
32	To what extent have you been trying to avoid any discussion about COVID-19 with family members?					
	During the COVID-19 pandemic, many of us have started taking action to cope with negative thoughts and emotions. How far do you think you have practiced these actions to cope with discomfort due to the COVID-19 pandemic? Please rate the same on a scale of 0-3. Please tick anyone of the following options					
33	Watching TV/videos/reading books					
34	Accessing and responding to social media involvement (WhatsApp, making videos, Facebook, Instagram, etc.)					
35	Praying and meditation					
36	Doing exercise and yoga					
37	Engaging in hobbies (like cooking, painting, singing, writing poetry, etc.)					
38	Drinking herbal products like green tea/Kadha					
